# Where to Retire? Experiences of Older African Immigrants in the United States

**DOI:** 10.1093/geroni/igab046.1756

**Published:** 2021-12-17

**Authors:** Manka Nkimbeng, Alvine Akumbom, Marianne Granbom, Sarah Szanton, Tetyana Shippee, Roland Thorpe, Joseph Gaugler

**Affiliations:** 1 University of Minnesota, Minneapolis, Minnesota, United States; 2 Johns Hopkins School of Nursing, Baltimore, Maryland, United States; 3 Centre of Ageing and Supportive Environments (CASE), Lund University, Skane Lan, Sweden; 4 Johns Hopkins University, Baltimore, Maryland, United States; 5 University of Minnesota, University of Minnesota, Minnesota, United States; 6 Johns Hopkins Bloomberg School of Public Health, baltimore, Maryland, United States

## Abstract

The needs and conceptualization of age-friendliness likely vary for immigrant older adults compared to native-born older adults. For example, Hispanic immigrant older adults often return to their home country following the development of ill health. Doubling in size since the 1970’s, the aging needs of African immigrants are not fully understood. This qualitative study examined experiences of aging and retirement planning for African immigrant older adults in the United States (U.S.). Specifically, it explored the factors, processes, and ultimate decision of where these older adults planned to retire. We analyzed semi-structured interviews with 15 older African immigrants in the Baltimore-Washington Metropolitan area. Data were analyzed using thematic analyses in NVivo. The majority of participants were women, with a mean age of 64. We identified three overarching themes with ten sub-themes. The themes included: 1) cultural identity: indicating participant’s comfort with the U.S. society and culture; 2) decision making: factors that impact participants' choice of retirement location, and 3) decision made: the final choice of where participants would like to retire. Age-friendliness for immigrant older adults in the U.S. is complex and it includes the traditional domains such as physical and sociocultural environment (e.g. housing, transportation, and income). However, immigrant age-friendliness also needs to include wider contextual aspects such as political climate in their country of origin, immigrant status, family responsibilities, and acculturation in the U.S. More research is needed understand and facilitate age-friendly environments for transnational immigrant older adults.

